# Active *Helicobacter pylori* Infection is Independently Associated with Nonalcoholic Steatohepatitis in Morbidly Obese Patients

**DOI:** 10.3390/jcm9040933

**Published:** 2020-03-30

**Authors:** Michael Doulberis, Simone Srivastava, Stergios A Polyzos, Jannis Kountouras, Apostolis Papaefthymiou, Jolanta Klukowska-Rötzler, Annika Blank, Aristomenis K Exadaktylos, David S Srivastava

**Affiliations:** 1 Department of Gastroenterology and Hepatology, University of Zurich, Zurich 8091, Switzerland; doulberis@gmail.com; 2 Second Medical Clinic, School of Medicine, Aristotle University of Thessaloniki, Ippokration Hospital, Thessaloniki 54642, Macedonia, Greece; jannis@auth.gr (J.K.); appapaef@hotmail.com (A.P.); 3 Emergency Department, University Hospital Inselspital, Bern 3010, Switzerland; jolanta.klukowska-roetzler@insel.ch (J.K.-R.); davidshiva.srivastava@insel.ch (D.S.S.); 4 First Laboratory of Pharmacology, School of Medicine, Aristotle University of Thessaloniki, Thessaloniki 54124, Macedonia, Greece; spolyzos@auth.gr; 5 Department of Gastroenterology and Hepatology, Spital Thun, Thun 3600, Switzerland; simone.srivastava@spitalstsag.ch; 6 Department of Gastroenterology, University Hospital of Larisa, Mezourlo, Larisa 41110, Greece; 7 Institute for Pathology, University of Bern, Bern 3012, Switzerland; annika.blank@pathology.unibe.ch

**Keywords:** nonalcoholic steatohepatitis, NASH, NAFLD, nonalcoholic fatty liver disease, *Helicobacter pylori*, *Hp*, metabolic syndrome, MetS

## Abstract

Nonalcoholic fatty liver disease (NAFLD) emerges as an important global burden and *Helicobacter pylori* infection (*Hp*-I) has been suggested as a risk **factor of **NAFLD, although controversy exists. This retrospective study aimed to investigate a potential impact of active *Hp*-I on NAFLD severity in morbidly obese patients, subjected to bariatric surgery and gastric biopsy for documentation of *Hp*-I. Of 64 eligible participants, 15 (23.4%) were diagnosed with active *Hp*-I, showing higher rates of nonalcoholic steatohepatitis (NASH) than those without *Hp*-I (86.7% vs. 26.5%, respectively; *p* < 0.001). Concerning histological lesions, steatosis grade (*p* = 0.027), ballooning (*p* < 0.001), lobular inflammation (*p* = 0.003), and fibrosis stage (*p* < 0.001) were also more severe in *Hp*-I positive patients. Likewise, liver function tests, insulin resistance, dyslipidemia, and arterial hypertension were significantly higher in *Hp*-I positive patients. *Hp*-I was independently positively associated with NASH (beta = 3.27; *p* = 0.002), severe NASH (beta = 2.37; *p* = 0.018), and the presence of fibrosis (beta = 3.86; *p* = 0.001) in a binary regression model, after adjustment for potential confounders. In conclusion, active *Hp*-Ι was independently associated with NASH and fibrosis, findings offering potential clinical implication.

## 1. Introduction

Nonalcoholic fatty liver disease (NAFLD), as the hepatic component of the metabolic syndrome (MetS), represents a leading cause of chronic liver disease; its estimated global prevalence is about 25% of the general population [1] whereas in morbidly obese individuals its prevalence might rise up to 90% [1,2]. NAFLD is bidirectionally linked to MetS and insulin resistance (IR) [3]. The general term NAFLD encompasses a spectrum of distinct pathologies; simple steatosis (nonalcoholic fatty liver, NAFL) represents a reportedly benign entity, but might progress in a percentage of patients after the effect of “multiple hits” [2,3,4], to nonalcoholic steatohepatitis (NASH), which may also progress to fibrosis, liver cirrhosis, and hepatocellular carcinoma [3].

One of the potential pathogenetic “multiple hits, is regarded to be Helicobacter pylori infection (*Hp*-I) [1], with an estimated prevalence of 58% (varying from 39.9%–91.7%) worldwide [5]. *Hp*-I exhibits pleiotropic local and systematic manifestations. Peptic ulcer, nonulcer dyspepsia, gastric adenocarcinoma, gastric mucosa-associated lymphoid tissue lymphoma, and colorectal cancer belong to its classical pathogenicity [6,7]. Beyond its established local pathologies, *Hp*-I has been associated with systematic entities, including MetS, IR, diabetes mellitus type 2 (T2DM), coronary artery disease, and neurodegenerative diseases [1,8]. In addition, there is accumulating evidence, that *Hp*-I might contribute to the development and progression of NAFLD, as concluded in several studies [9,10,11,12,13], although controversy still exists [14,15,16], as we have previously systematically reviewed [1]. Notably, meta-analyses, having been conducted on the association between *Hp*-I and NAFLD, showed higher rates of NAFLD in *Hp* infected individuals, as we have also reviewed [5].

Together with the aforementioned high prevalence of NAFLD in morbidly obese individuals, the prevalence of *Hp* seropositivity in these patients is also higher (a 1.7-fold increased probability of having *Hp*-I) compared with nonobese controls [17]. However, the serological test does not discriminate between active and past infection. Beyond serological testing for *Hp*-I, there are further diagnostic modalities used for the investigation of a possible association between *Hp*-I and NAFLD: Urea breath test [18,19,20,21,22,23] and the detection of *Hp* antigen in patients’ stool [24] for the diagnosis of *Hp*-I, as well as sonography [10,19,23,24,25,26], and liver function tests and various noninvasive indices [10,11,22,27] for the diagnosis of NAFLD. Nevertheless, ^13^C urea breath and *Hp* antigen fecal tests are also not sufficient to substitute the histological diagnostic “gold standard” for detection of active *Hp*-I; the gold standards for the diagnosis of *Hp*-I and NAFLD are currently histological examination after gastric and liver biopsies, respectively [28,29,30,31]. However, studies with gastric and liver biopsies are currently scarce [14].

The aim of this study was to investigate a potential association of active *Hp*-I with NAFLD and its histological severity in morbidly obese patients subjected to bariatric surgery, gastric, and liver biopsy.

## 2. Methods

### 2.1. Study Design

The study was performed retrospectively, using an electronic database with 94,304 case records of patients who have been presented to the emergency department (ED) of Inselspital Bern from 1 January 2017 to 30 November 2018. This academic hospital offers its medical services to an area of approximately 2,000,000 citizens.

### 2.2. Ethical Considerations

The last revision of the principles of the Declaration of Helsinki was fulfilled. The study was approved by the cantonal (district) ethics committee in Berne, (Kantonale Ethikkommission Bern, Ref. No. KEK-BE: 010/2016) and since our patients were fully anonymized prior to analysis, according to Swiss law, informed consent was not mandatory.

### 2.3. Inclusion and Exclusion Criteria

Eligible were morbidly obese patients older than 16 years having been subjected to bariatric surgery, as well as to liver and gastric biopsy for documentation of NAFLD and active *Hp*-I, respectively. Exclusion criteria were: Ethanol consumption (>20 g/day); previous *Hp* eradication treatment; known history of other active or past hepatobiliary disease; history of gastrectomy; malignancies; inappropriate thyroid or adrenal function; kidney disease; pregnancy or lactation; use of glucocorticoids, insulin, orlistat, methotrexate, amiodarone, vitamin E, pioglitazone, intravenous glucose, or parenteral nutrition administration; any drug addiction; type 1 DM; pancreatitis; thrombotic disorders. Patients with T2DM were excluded if they were on thiazolidinediones or insulin treatment.

### 2.4. Data Collection and Extraction 

Morbidly obese patients undergoing an elective bariatric operation were recruited. Specifically, the *Hp*-I status was evaluated by the diagnostic “gold standard” histology from gastric biopsies, obtained either preoperatively (in terms of a routine esophagogastroduodenoscopy) or intraoperatively. Histological diagnosis of *Hp* gastritis was made on 5 μm formalin-fixed, paraffin-embedded tissue sections by means of Hematoxylin and Eosin staining. A modified Giemsa stain was also used to highlight the *Hp* bacteria.

Liver specimens were obtained intraoperatively during laparoscopic bariatric surgery, from liver segment II or, less commonly, segment III. Grading and staging of NAFLD was based on the NASH Clinical Research Network scoring system [32]; for the discrimination between NAFL and NASH, both NAFLD activity score (NAS) [32] and fatty liver inhibition of progression (FLIP) were used, the latter introduced in morbidly obese populations [29]. Severe NASH was defined as steatosis, activity, and fibrosis (SAF) scoring system ≥3 and/or F ≥ 3, as elsewhere recommended [33]. 

Patients’ records at the time of admission to the ED were stored in the clinical application E.care for Windows (E.care BVBA, ED 2.1.3.0, Turnhout, Belgium). E.care offers the advantage of instantaneous recall of medical reports, and other relevant data, while multiple filters of E.care application can be applied. Patients’ records from ED were extracted to an Excel sheet (Microsoft^®^ Excel for Mac 2019, Microsoft Corporation, Redmond, WA, USA) with the use of appropriate filters. Eligibility of the retrieved patients was evaluated by two investigators (M.D. and S.S.), following the inclusion and exclusion criteria. Selected data were validated by D.S.S. In cases of conflict, a consensus was met by the intervention of a senior author (A.Ε.). The following parameters were extracted: (a) Demographics and anthropometric (age, gender, body-mass index (BMI)); (b) histological findings of gastric and liver biopsies: *Hp*-I positivity, steatosis grade, lobular inflammation, ballooning degeneration, and fibrosis; (c) MetS components, i.e., IR, dyslipidemia, hypertension, and T2DM, as recommended by the Expert Committee on the Diagnosis and Classification of Diabetes [34]; in this regard, dyslipidemia was defined as triglyceride levels ≥150 mg/dL (1.7 mmol/L) or LDL-C levels ≥ 100–160 mg/dl (2.58–4.13 mmol/L, depending on other risk factors) or HDL-C levels < 40 and 50 mg/dl (1.03 and 1.29 mmol/L) in men and women, respectively, or treatment with hypolipidemic medication(s); arterial hypertension was defined as systolic blood pressure ≥ 130 mmHg or diastolic blood pressure ≥ 85 mmHg or treatment with antihypertensive medication(s); (d) laboratory tests: Fasting glucose and fasting insulin (for the calculation of IR, using the homeostasis model assessment IR (HOMA-IR) [35]), glycated hemoglobin A1c (Hba1c), liver function tests (aspartate aminotransferase (AST), alanine aminotransferase (ALT), gamma-glutamyl transferase (GGT), bilirubin).

### 2.5. Statistical Analysis

Data are presented as mean ± standard deviation (SD) and percentages, for continuous and categorical variables, respectively. The normality of distributions of continuous variables was tested with the Kolmogorov–Smirnov test. The comparisons of continuous and categorical variables were performed with the Mann–Whitney test and chi-square test (or the Fischer exact test), respectively. Binary logistic regression analysis was performed to investigate whether *Hp*-I was independently associated with NASH, severe NASH and fibrosis, because they are considered the main histological endpoints. As independent variables, we entered parameters related to MetS (BMI, hypertension, dyslipidemia, HOMA-IR), as well as age and gender. Statistical analysis was performed with SPSS 21.0 for Macintosh (IBM Corp., Armonk, NY, USA). Significance was set at *p* < 0.05 (two tailed).

## 3. Results

Sixty-four patients (47 women), subjected to both gastric and liver biopsy, were recruited in this study. Fifteen (23.4%) patients were positive for *Hp*-I. According to the FLIP algorithm, nine (14.1%) patients were classified as “No NAFLD”, 29 (45.3%) as NAFL, and 26 (40.6%) as NASH. Severe NASH was observed in 13 (20.3%) patients. According to NAS, eight (12.5%) patients were classified as “No NAFLD”, 25 (39.0%) as NAFL, 17 (26.6%) as borderline NASH, and 14 (21.9%) as definite NASH. Regarding fibrosis stage, 39 (61.0%) patients were at F0, 18 (28.1%) at F1, 5 (7.8%) at F2, 2 (3.1%) at F3, whereas none were at F4; due to the small number of patients at F3, categories F2 and F3 were merged for the analysis (F2/3: 7 (10.9%) patients).

**Figure 1:**
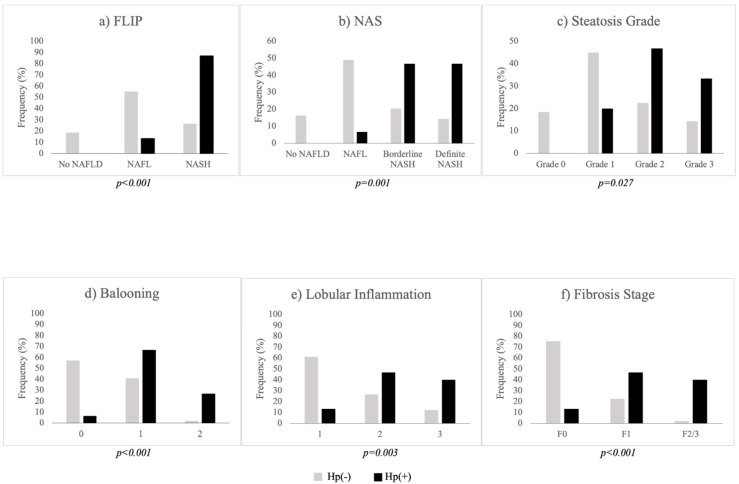
Bar grams depicting the histological endpoints: (**a**) FLIP; (**b**) NAS; (**c**) steatosis grade; (**d**) ballooning; (**e**) lobular inflammation; (**f**) fibrosis stage. The rates of *Hp* infection were increased with the severity of all presenting histological lesions and composite scores (FLIP and NAS). *Hp*(+): Black bars; *Hp*(−): Grey bars. Abbreviations: F: Fibrosis stage; FLIP: Fatty liver inhibition of progression; *Hp*(+): *Helicobacter pylori* positive; *Hp*(−): *Helicobacter pylori* negative; NAFLD: Nonalcoholic fatty liver disease; NAS: Nonalcoholic fatty liver disease activity score; NASH: Nonalcoholic steatohepatitis.

**Figure 2:**
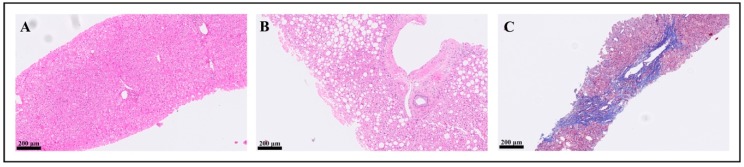
Liver microphotographs of patients with the most representing stages of nonalcoholic fatty liver disease. (**A**) Formalin fixed, paraffin embedded histological section (hematoxylin and eosin staining) of liver with normal architecture. (**B**) Formalin fixed, paraffin embedded histological section (hematoxylin and eosin staining) of liver with prominent steatosis without activity or fibrosis. (**C**) Formalin fixed, paraffin embedded histological section (Masson trichrome staining) of liver with bridging fibrosis.

Comparative data between *Hp*-I positive [*Hp*(+)] and negative [*Hp*(*−*)] patients are presented in Table 1. *Hp*(+) and Hp(−) groups were of similar gender age, and BMI. AST, ALT, GGT, bilirubin, triglycerides, and HOMA-IR were higher in *Hp*(+) than in *Hp*(−) group, whereas total cholesterol, HDL-C, and LDL-C were not statistically different between groups. Higher rates of hypertension (*p = 0.041*) and triglyceridemia (*p = 0.039*), but not prediabetes/diabetes, were observed in *Hp*(+) than *Hp*(−) group. Regarding both FLIP and NASH classifications, higher rates of NASH were observed in *Hp*(+) than in *Hp*(−) group (Table 1). Likewise, higher rates of severe NASH were observed in *Hp*(+) than in *Hp*(−) group. Concerning separate histological lesions, histological severity was more prominent in *Hp*(+) than *Hp*(−) patients in steatosis grade, ballooning, lobular inflammation, and fibrosis (Table 1, Figure 1). Representative histological images from the included patients are illustrated in Figure 2. When the analysis was repeated in women (n = 47), the results remained essentially unchanged. However, the analysis in men was avoided, mainly owing to the small sample of men (n = 17), which might have led to insecure conclusions. 

Binary regression analysis was performed with FLIP as dependent variable in a subset of patients (NALF vs. NASH; n = 55), after the exclusion of those without NAFLD (n = 9). The results of this analysis are presented in Table 2. NASH remained independently positively associated with *Hp*-I (*p* = 0.002) after adjusting for potential confounding. Next, regression analysis was performed with severe NASH as dependent variable (No vs. Yes) in the sum of patients. Again, severe NASH remained independently positively associated with *Hp*-I (*p* = 0.018; Table 3). In this model, age and BMI were also independently positively associated with severe NASH. Likewise, when fibrosis (F0 vs. F1-3) in the sum of patients was selected as dependent variable, the presence of fibrosis remained independently positively associated with *Hp*-I (*p* = 0.001; Table 4). In this model gender was also independently associated with the presence of fibrosis (i.e., men had independently higher risk of fibrosis).

## 4. Discussion

This study favors an association between active *Hp*-I and histological severity of NAFLD in morbidly obese patients subjected to bariatric surgery. Specifically, higher rates of NASH, as well as hepatic steatosis, inflammation, and fibrosis were observed in *Hp*(+) than *Hp*(−) patients, findings warranting further investigation. Notably, *Hp*-I was independently positively associated with NASH, severe NASH, and hepatic fibrosis, the latter considered as the main histological prognostic factor [36].

Importantly, the histological diagnostic “gold standard” for both main variables of interest (active *Hp*-I and NAFLD) were used, which is scarce in the literature. More specifically, this is the second study, after the study of Lecube et al. [14] investigating the association between *Hp*-I and NAFLD by using both gastric and liver biopsies, in which liver biopsy was performed in a subset of patients (22% of patients subjected to gastric biopsy). In that study, higher steatosis rates were observed in *Hp*(+) patients, as in our study, but, unexpectedly rates of NASH were higher in *Hp*(−) patients. Moreover, contrary to our finding, the authors reported similar overall NAFLD rates between *Hp*(+) and *Hp*(*−*) patients [14]. There is no secure conclusion about the controversy of our with Lecube et al. [14] study; population and/or methodological differences may exist, thus warranting more studies, specifically designed to this aim. 

Noteworthy, we were the first to show an association between *Hp*-I and NAFLD in a case-control study of biopsy-proven NAFLD patients [37]. Subsequently, other studies reported a positive association between *Hp*-I and NAFLD, including those of Yu et al. [19], Sumida et al. [38], Okushin et al. [18], Kim et al. [10,27], Zhang et al. [21], Chen et al. [26], Dogan et al. [25], Abdel-Razik et al. [24], and Kang et al. [11]. Noteworthy, there have been five systematic reviews performed with meta-analysis, all of which revealed a positive association between the pathologies of interest [39,40,41,42,43]. Nonetheless, there are studies showing no association between *Hp*-I and NAFLD [14,15,19,20,22].

The association between *Hp*-I and components related to IR or MetS were also investigated in this study as secondary aims. Higher liver function tests, triglycerides, HOMA-IR, and higher rates of arterial hypertension were observed in *Hp*(+) compared with *Hp*(−) patients. In a systematic review we performed almost 10 years ago, we have proposed a positive association between *Hp*-I and IR, in terms of HOMA-IR [44]. Although controversy still exists, similar results to this study have provided a meta-analysis between *Hp*-I, MetS, and MetS-related comorbidities [45].

The observational nature of this study cannot show causality or direction of the relationship, i.e., to answer the question whether *Hp*-I is another pathogenetic factor of NAFLD or, inversely, NAFLD makes affected individuals more susceptible to *Hp*-I. However, pathogenetic mechanisms between *Hp*-I and NAFLD may be hypothesized. *Hp*-I related gastrointestinal epithelium disruption, microbiota, and their metabolites translocation into portal circulation, activation of inflammation via toll-like receptors signaling in hepatocytes, may be some contributors linking *Hp*-I with the development and progression of NAFLD [3,46]. In this regard, human β-defensin-1, possibly serving as a biomarker of bacterial translocation in chronic liver disease, is induced by *Hp*-I. *Hp*-I also contributes to a low-grade inflammation by inducing the release of vasoactive and proinflammatory molecules, including interleukin (IL)-1, IL-6, IL-8, IL-10, IL-12, tumor necrosis factor-α, interferon-γ, eicosanoids (leukotrienes, prostaglandins), and acute phase proteins (C-reactive protein and fibrinogen), which are implicated in the pathophysiology of IR syndrome and NAFLD. Moreover, *Hp*-related induction of oxidative stress, atrophic gastritis-associated with vitamin B12/folate deficiency, apoptosis, and the possible downregulation of adiponectin, constitute other pathogenetic links between *Hp*-I and NAFLD [3,46]. Furthermore, *Hp*-related galectin-3 connected with MetS may play a role in the pathophysiology of MetS-related NAFLD and its extrahepatic complications, including chronic kidney, cardiovascular, and brain disorders; the number of galectin-3 positive liver cells is associated with NAFLD severity; galectin-3 binding protein is a potential marker of disease; and galectin-3 inhibitors may exhibit potential efficacy in advanced fibrosis/cirrhosis due to NASH. Finally, *Hp*-related MetS conditions are associated with dysmotility-induced microbiota dysbiosis, particularly small intestinal bacterial overgrowth (SIBO) playing a role in the pathogenesis of NAFLD; SIBO exhibits an effect on the structural and functional issues of the liver leading to greater prevalence of NAFLD. Notably, SIBO prevalence is higher in morbidly obese patients than in healthy populations and is connected with severe hepatic steatosis [47]. However, further large-scale relative studies are needed to elucidate in depth the aforementioned proposed pathogenetic mechanisms.

Although the use of both liver and gastric biopsies is a strength of this study, there are also certain limitations. First, the retrospective nature of the study renders it susceptible to record and recall bias. Second, as mentioned above, a cause–effect relationship cannot be shown. Third, the sample size was small, although it was sufficient to show robust statistical significance in major endpoints. Furthermore, information on previous use of proton pump inhibitors before obtaining gastric biopsies was not available. Last, the duration of *Hp*-I, which might have affected the results, remains unknown.

## 5. Conclusions

This study favors an association of active *Hp*-I on NAFLD severity in morbidly obese patients subjected to bariatric surgery. Most importantly, active *Hp*-I was independently associated with NASH and the presence of fibrosis. These findings warrant specifically designed clinical studies on the association between active *Hp*-I and NAFLD severity in obese, morbidly obese, and lean populations, as well as mechanistic studies investigating the potential pathogenetic links.

## Figures and Tables

**Table 1:** Comparative data between *Hp*(+) and *Hp*(−) patients.

	*Hp*(−) N = 49 (76.6%)	*Hp*(+) N = 15 (23.4%)	*p*-Value *
**Women [N (%)]**	37 (75.5)	10 (66.7)	0.52
**Age (years)**	46.7 ± 13.3	49.1 ± 12.4	0.51
**BMI (kg/m^2^)**	45.5 ± 10.2	42.6 ± 5.9	0.70
**AST (U/L)**	24.5 ± 9.0	42.7 ± 18.9	**0.001**
**ALT (U/L)**	28.6 ± 20.5	51.6 ± 24.5	**<0.001**
**GGT (U/L)**	35.7 ± 43.9	86.3 ± 60.2	**<0.001**
**Bilirubin (μmol/L)**	7.8 ± 5.2	12.2 ± 8.3	**0.013**
**Triglycerides (mmol/L)**	1.48 ± 1.58	2.02 ± 0.98	**0.003**
**Total cholesterol (mmol/L)**	4.39 ± 1.14	4.79 ± 0.46	0.10
**HDL-C (mmol/L)**	1.29 ± 0.37	1.23 ± 0.46	0.55
**LDL-C (mmol/L)**	2.48 ± 0.85	2.72 ± 0.64	0.20
**HbA1c (%)**	5.7 ± 0.6	6.0 ± 1.3	0.33
**HOMA-IR**	4.3 ± 3.9	10.1 ± 6.8	**<0.001**
**Prediabetes/Diabetes [N (%)]**	24 (49.0)	9 (60.0)	0.46
**Arterial hypertension [N (%)]**	18 (36.7)	10 (66.7)	**0.041**
**FLIP [N (%)]**			**<0.001**
**No NAFLD**	9 (18.4)	0 (0.0)	
**NAFL**	27 (55.1)	2 (13.3)	
**NASH**	13 (26.5)	13 (86.7)	
**Severe NAFLD [N (%)]**	6 (12.2)	7 (46.7)	**0.008**
**NAS [N (%)]**			**0.001**
**No NAFLD**	8 (16.3)	0 (0.0)	
**NAFL**	24 (49.0)	1 (6.6)	
**Borderline NASH**	10 (20.4)	7 (46.7)	
**Definite NASH**	7 (14.3)	7 (46.7)	
**Steatosis [N (%)]**			**0.027**
**Grade 0**	9 (18.4)	0 (0.0)	
**Grade 1**	22 (44.9)	3 (20.0)	
**Grade 2**	11 (22.4)	7 (46.7)	
**Grade 3**	7 (14.3)	5 (33.3)	
**Ballooning [N (%)]**			**<0.001**
**0**	28 (57.1)	1 (6.6)	
**1**	20 (40.8)	10 (66.7)	
**2**	1 (2.0)	4 (26.7)	
**Lobular inflammation [N (%)]**			**0.003**
**0**	30 (61.2)	2 (13.3)	
**1**	13 (26.5)	7 (46.7)	
**2**	6 (12.2)	6 (40.0)	
**Fibrosis stage [N (%)]**			**<0.001**
**F0**	37 (75.5)	2 (13.3)	
**F1**	11 (22.4)	7 (46.7)	
**F2/3**	1 (2.0)	6 (40.0)	

Data are presented as mean ± standard deviation (SD) for continuous, and frequencies (percentage) for categorical variables. * Between group comparisons (Mann–Whitney test for continuous and chi-square or Fischer exact test for categorical variables). Abbreviations: ALT: Alanine transaminase; AST: Aspartate transaminase; BMI: Body mass index; FLIP: Fatty liver inhibition of progression; GGT: Gamma-glutamyl transferase; HbA1: Glycated hemoglobin; HDL: High density lipoprotein; HOMA-IR: Homeostatic model of assessment insulin resistance; LDL: Low density lipoprotein; NAFLD: Nonalcoholic fatty liver disease; NAS: Nonalcoholic fatty liver disease activity score; NASH: Nonalcoholic steatohepatitis.

**Table 2:** Independent associates of NASH (NAFL vs. NASH), according to FLIP classification in binary logistic regression analysis.

Independent Variables	Beta	Exp(Beta)	*p*-Value	95% CI for Exp(Beta)
***Hp*-I diagnosis** **(0: Negative; 1: Positive)**	3.27	26.32	0.002	3.36–206.23
**Gender** **(0: Women; 1: Men)**	1.30	3.68	0.14	0.65–20.73
**Hypertension** **(0: No; 1: Yes)**	−1.17	0.31	0.19	0.05–1.80
**Hyperglyceridemia** **(0: No; 1: Yes)**	−0.33	0.72	0.70	0.14–3.75
**Age (years)**	0.03	1.03	0.40	0.97–1.09
**BMI (kg/m^2^)**	0.05	1.06	0.15	0.98–1.14
**HOMA-IR**	0.15	1.17	0.12	0.96–1.41

Abbreviations: BMI: Body mass index; FLIP: Fatty liver inhibition of progression; HOMA-IR: Homeostatic model of assessment insulin resistance; *Hp*-I: *Helicobacter pylori* infection; NAFL: Nonalcoholic fatty liver; NASH: Nonalcoholic steatohepatitis.

**Table 3:** Independent associates of severe NASH (No vs. Yes), according to FLIP classification in binary logistic regression analysis.

Independent Variables	Beta	Exp(Beta)	*p*-Value	95% CI for Exp(Beta)
***Hp*-I diagnosis** **(0: Negative; 1: Positive)**	2.37	10.73	0.018	1.50–76.46
**Gender** **(0: Women; 1: Men)**	0.06	1.06	0.95	0.17–6.58
**Hypertension** **(0: No; 1: Yes)**	0.06	1.07	0.94	0.20–5.74
**Hyperglyceridemia** **(0: No; 1: Yes)**	0.44	1.56	0.62	0.27–9.09
**Age (years)**	0.09	1.10	0.028	1.01–1.19
**BMI (kg/m^2^)**	0.10	1.10	0.035	1.01–1.21
**HOMA-IR**	0.02	1.02	0.82	0.88–1.17

Abbreviations: BMI: Body mass index; FLIP: Fatty liver inhibition of progression; HOMA-IR: Homeostatic model of assessment insulin resistance; *Hp*–I: *Helicobacter pylori* infection; NAFL: Nonalcoholic fatty liver; NASH: Nonalcoholic steatohepatitis.

**Table 4:** Independent associates of fibrosis (F0 vs. F1-3) in binary logistic regression analysis.

Independent Variables	Beta	Exp(Beta)	*p*-value	95% CI for Exp(Beta)
***Hp*-I diagnosis** **(0: Negative; 1: Positive)**	3.86	47.28	0.001	4.76–469.5
**Gender** **(0: Women; 1: Men)**	1.95	7.00	0.026	1.26–39.02
**Hypertension** **(0: No; 1: Yes)**	−0.18	0.84	0.82	0.18–3.90
**Hyperglyceridemia** **(0: No; 1: Yes)**	−0.20	0.82	0.81	0.16–4.11
**Age (years)**	0.05	1.05	0.08	0.99–1.12
**BMI (kg/m^2^)**	−0.05	0.95	0.37	0.86–1.06
**HOMA-IR**	−0.04	0.96	0.56	0.83–1.11

Abbreviations: BMI: Body mass index; FLI: Fatty liver inhibition of progression; HOMA-IR: Homeostatic model of assessment insulin resistance; *Hp*-I: *Helicobacter pylori* infection; NAFL: Nonalcoholic fatty liver; NASH: Nonalcoholic steatohepatitis.

## Abbreviations

BMI: Body-mass index; ED: Emergency department; FLIP: Fatty Liver Inhibition of Progression; GI: Gastrointestinal; Hba1: Glycated hemoglobin A1c; HOΜA-IR: Homeostatic model of assessment insulin resistance; *Hp*: *Helicobacter pylori; Hp*-I: *Helicobacter pylori* infection; IR: Insulin resistance; IL: Interleukin; MetS: Metabolic syndrome; NAFLD: Nonalcoholic fatty liver disease; NAS: NAFLD activity score; NASH: Nonalcoholic steatohepatitis; SAF: Steatosis, activity, and fibrosis; SIBO: Small intestinal bacterial overgrowth; T2DM: Diabetes mellitus type 2.
